# Assessing Physician Confidence in Counseling Patients on COVID-19 Disease and Vaccination: A Survey of Physicians’ Perspectives in the Kingdom of Saudi Arabia

**DOI:** 10.7759/cureus.52887

**Published:** 2024-01-24

**Authors:** Saja S Alerwi, Abdulaziz Z Alqasir, Hussain Alzahrani, Laalie M Hafiz, Maysoon Alharthy, Mohammed Albalawi, Renad A Alhazmi, Roba A Alhazmi, Saad G Alotaibi, Saad Alsaleh, Yomna K Alahmadi, Khaldoon A Alroomi

**Affiliations:** 1 College of Medicine, Arabian Gulf University, Manama, BHR; 2 Family Medicine, Arabian Gulf University, Manama, BHR

**Keywords:** adverse effects, patients’ confidence, consultation, vaccines, covid-19 pandemic

## Abstract

Background: The COVID-19 vaccine has been available and well acceptable among the Saudi population since its introduction in 2020; however, concerns still remain regarding the confidence of doctors in giving such vaccines.

Research question: How confident are physicians in the Kingdom of Saudi Arabia about giving counseling and advice for patients regarding COVID-19 disease and COVID-19 vaccines?

Objective: To determine how confident Saudi Arabian doctors are in their ability to advise patients on the COVID-19 illness and COVID-19 vaccines.

Aim: To assist the health authorities in the Kingdom of Saudi Arabia in developing and implementing programs to improve doctors’ skills and knowledge in giving advice to patients about the COVID-19 vaccine.

Method: The research employed an online cross-sectional study methodology to gather insights from doctors employed at hospitals, healthcare centers, and clinics across the Kingdom of Saudi Arabia. The inclusion criteria encompassed doctors actively engaged in healthcare settings, while the exclusion criteria were applied to those who had never encountered COVID-19 patients and those who declined participation in the study.

Results: It was found that doctors were confident that there’s a potential for adverse effects that are not yet seen in vaccine trials. Further results showed that primary health care doctors are more confident about the common side effects associated with the approved vaccines.

Conclusion: Most doctors were confident about the vaccine, yet they also know that there are some hidden side effects that are not yet discovered. Since patients trusted doctors as the main source of information about the vaccine, the study provided evidence to emphasize the rule of doctors as a reliable source of information.

## Introduction

Coronavirus illness (COVID-19) is a virus infection caused by the SARS-CoV-2 virus that was discovered in 2019. SARS-CoV-2 was initially identified in Wuhan, China. The WHO has categorized COVID-19 as a pandemic since it expanded extensively in the first half of 2020 [1]. On January 23, 2023, the total documented coronavirus cases in Saudi Arabia were 827,658 with a total fatality of 6,746,918 [2]. The Pfizer-BioNTech vaccine was the first vaccine to be approved against COVID-19. In Saudi Arabia, four vaccinations have been approved for use [3]. Moderna and Pfizer-BioNTech are RNA-based vaccinations that contain viral genetic material that produces viral protein, while Janssen (Johnson & Johnson) and Oxford-AstraZeneca are non-replicating viral vector vaccines that pack viral genetic material inside a harmless virus [4]. On December 11th, 2020 the Saudi Food and Drug Authority (SFDA) authorized Pfizer-BioNTech, while Oxford-AstraZeneca and Moderna were approved on February 18th and July 9th, 2021, respectively [5,6]. The Saudi Ministry of Health estimates that 48.330.859 vaccination doses will be administered till December 19th, 2021. 64.82% of the Saudi population has been fully immunized [7].

Pertinent materials or information and related studies on the topic or subject of COVID-19 vaccination are discussed and elaborated from literature and internet sources accessed. Announcements declaring promising interim results of multiple candidate COVID-19 vaccines, combined with governmental authorization for their use in mass vaccination programs, offer a glimmer of hope for a potential return to normalcy. Numerous patients have valid inquiries regarding the safety, effectiveness, and acceptance of these vaccines and frequently turn to their general practitioner (GP) for answers. GPs are seen as trustworthy sources of vaccine information and are at the forefront of vaccine administration, making them easily accessible to address these concerns [8].

According to a recent article published in the Journal of the American Medical Association (JAMA) in 2021, there has been a concerning decline in the willingness of people to get vaccinated. The percentage of individuals who expressed being somewhat or very likely to get vaccinated decreased from 74% in April 2020 to 56% in December 2020 [9]. This decrease in vaccine confidence is alarming for all of us. On a positive note, primary care physicians enjoy high levels of trust from the public. A recent survey conducted by NPR/Ipsos revealed that 85% of respondents trust their personal physician when asked about their level of trust towards different individuals. This trust in physicians remained consistent regardless of political affiliation, with 84% of Republicans, 89% of Democrats, and 86% of independents expressing trust in "their physician" [10]. The Wellcome Global Monitor survey (2018) stated that only one-quarter of respondents globally expressed a lot of trust in their government, and fewer than half of respondents globally said that they trust doctors and nurses a lot. People's trust in these institutions was correlated with trust in health or medical advice from them and with more positive attitudes toward vaccines [11].

The main goal of our study is to investigate how doctor-patient communication influences vaccination behavior. We examine the role of doctor-patient communication in building trust in doctors' vaccine recommendations, which in turn leads to positive attitudes towards vaccination and ultimately results in actual H1N1 vaccination. These findings carry important implications for public health organizations in terms of integrating effective vaccine communication. Moreover, they could have crucial implications for the COVID-19 vaccination efforts [12]. The major highlight of this study went hand in hand with the study conducted in Yemen, which stated that HCWs had overall good knowledge, suboptimal preparedness, and adequate counseling practices prior to the outbreak of COVID-19 in Yemen, despite the high number of perceived barriers. However, urgent action and interventions are needed to improve the preparedness of HCWs to manage COVID-19. The perceived barriers also need to be fully addressed by the local healthcare authorities and international organizations working in Yemen for adequate prevention and control measures to be in place in managing COVID-19 [13].

In contrast to the conflicting guidance from government agencies and professional associations, evidence-based professional ethics in obstetrics and gynecology provides unequivocal and clear guidance. To prevent the widening of health inequities, build trust in the health benefits of vaccination, and encourage coronavirus disease 2019 vaccine and treatment uptake, in addition to recommending coronavirus disease 2019 vaccinations, physicians should engage with communities to tailor strategies to overcome mistrust and deliver evidence-based information, robust educational campaigns, and novel approaches to immunization [14].

In conclusion, most hospitalists would reportedly advise patients to receive the COVID-19 vaccine. Hospitalists are an important resource to provide patient education and reduce COVID-19 vaccine hesitancy. In order to accomplish this, we must raise public awareness of the value of vaccinations, clearly explain their efficacy and safety, respond to individual worries, and foster a climate of trust surrounding the COVID-19 vaccine. Establishing trustworthy procedures to recognize, rank, and alert patients according to their age, disease risk, and location is also necessary. At the point of care, we also need to create a dependable, real-time patient tracking system [10].

## Materials and methods

Type of study and study population

An online cross-sectional study was conducted to obtain information from doctors who work at hospitals, healthcare centers, and clinics in the Kingdom of Saudi Arabia.

Inclusion criteria

Doctors who work at hospitals, healthcare centers, and clinics. 

Exclusion criteria

The study excluded doctors who have never dealt with COVID-19 patients and those who refused to participate.

Study variables

The study collected sociodemographic data for the doctors, including age, gender, specialty, years of experience, workplace, dealing with COVID-19 patients, and counseling patients about the COVID-19 vaccine. Age was categorized into three groups: 25-34, 35-44, and 45 years old and above. Gender was divided into male and female. Specialties included surgeon, internist, pediatrician, family physician, general physician, and others. Years of experience were classified into three categories: 1-10, 11-20, and 21 years and above. The workplace was categorized into five regions: north region, south region, west region, east region, and central region. Dealing with COVID-19 patients was divided into yes and no, and counseling patients about the COVID-19 vaccine was also divided into yes and no.

The response variable was the knowledge of doctors about COVID-19 disease. The questionnaire included information about their knowledge of the COVID-19 vaccine, the safety of the vaccine, and the effectiveness of the vaccine.

Data source and collection

The population was collected from hospitals in Saudi Arabia between June 22 and August 22, 2022.

The estimated sample size is:

N = (Z^2^) × P (1−P)/E^2^,

N = (1.96^2^) × 0.5 (1−0.5)/(0.05^2^ = 384−400),

where N is the sample size, E is the margin of error, Z is the critical value, and P is the prevalence. 

Study instrument

The collection of data was collected through electronic/online questionnaires using Google Forms that were available in English for doctors. 

Statistical analysis

After the data collection phase of the study is completed, the data is coded and entered into the computer. A standard computer analysis program, namely IBM SPSS Statistics for Windows, Version 20 (Released 2011; IBM Corp., Armonk, New York), is used to produce descriptive analysis, which includes frequency distribution tables, proportions, percentages, means, and standard deviations. The questionnaire is based on a Likert scale from 1-5; 1 is not satisfied and 5 is very satisfied. In inferential statistics, the chi-square test was used to analyze qualitative variables. 

Ethical considerations

The research proposal was submitted for review and approval by the Ethical Committee of the College of Medicine and Medical Science at Arabian Gulf University (Approval number: E5-PI-4-22/Group5). The information that we are going to acquire from participants was classified, and they have the right to agree or disagree on taking the survey. Their information was confidential, and the results were used only for scientific purposes. 

Research pilot study

We were initially producing an initial questionnaire that was submitted to 10 doctors; eventually, a final questionnaire was produced for all the groups in Saudi Arabia.

## Results

Table 1 represents the sociodemographic characteristics of the doctors. Regarding age, the percentage of respondents ranging between 25 and 34 years scored the highest percentage of 74.9%, whereas male respondents scored the highest percentage of 63.9%. Saudi national respondents were the majority with 77.9%. Primary health care doctors scored 57.9%, followed by secondary health care doctors with 30.6%. It was revealed that patient contact was not needed on an everyday basis, with a percentage of 53.6%. The table shows that the majority of respondents had ≤10 years of experience, with a percentage of 81% (Table 1). 

**Table 1:** Demographic characteristics of respondents.

Sociodemographic characteristics	
	n (%)
Age	
25-34 years	299 (74.9)
35-44 years	73 (18.3)
≥45 years	27 (6.8)
Gender	
Female	144 (36.1)
Male	255 (63.9)
Nationality	
Saudi	311 (77.9)
Non-Saudi	88 (22.1)
Specialty	
Primary health care	231 (57.9)
Secondary health care	122 (30.6)
Others	46 (11.5)
Patient contact	
Everyday	185 (46.4)
Not everyday	214 (53.6)
Years of experience	
≤10 years	323 (81)
>10 years	76 (19)
Workplace	
North region	19 (4.8)
South region	16 (4)
West region	128 (32.1)
East region	37 (9.3)
Central region	199 (49.9)

The analysis of the results in Table 2 related to the confidence of the doctors in their ability to counsel patients showed the following. The rate of confidence in vaccine safety is believed to have common side effects associated with the approved vaccine(s) and the presence of potential adverse effects that are not yet seen in vaccine trials with 62.7% and 45.1%, respectively. Regarding vaccine efficiency, doctors were confident about the efficacy of approved vaccine(s) for specific groups with 52.6%. Both the overall reported efficacy of approved vaccine(s) and the number of doses required to achieve this efficacy scored similar confidence rates by doctors with 51.6%. The efficacy of approved vaccine(s) refers to specific complications, and the potential benefits to patients who have previously been infected with COVID-19 were similarly trusted by doctors with 51.1%. The last item with the lowest confidence rate was the potential need for future boosters with 74.9%. Furthermore, the vaccine eligibility and prioritization in terms of the rationale for this priority list scored the highest confidence rate of 52.6%, followed by the order in which groups will be offered an approved vaccine with 50.4%. In regard to the practicalities of delivering vaccines to patients, the methods to offer an approved vaccine to eligible patients were much trusted by doctors with 56.4%. The results showed that the rate of confidence in the ability of patients to choose which approved vaccine they receive was 45.6% in regard to vaccine administration by doctors. It was found that patients were confident in the case where they should continue to follow public health guidance after receiving an approved vaccine in regard to the after-vaccine administration with 50.4%. Finally, the rate of confidence in the instances of vaccine misinformation (including anti-vax conspiracy theories) scored 43.9% (Table 2). 

**Table 2:** Level of confidence of doctors in their ability to counsel patients about taking vaccines. GP: general practitioner.

Level of confidence of doctors			
	Confident	Not sure	Not confident
	N = 399 (100%)	N = 399 (100%)	N = 399 (100%)
Vaccine safety			
Common side effects associated with approved vaccine(s)	250 (62.7)	31 (7.8)	118 (29.6)
Safety profile of approved vaccine(s) for specific groups (e.g., over 80 years old, co-morbidities, pregnancy, children)	217 (54.4)	112 (28.1)	70 (17.5)
The potential for adverse effects not yet seen in vaccine trials	180 (45.1)	113 (28.3)	106 (26.6)
Vaccine efficiency			
Overall reported efficacy of approved vaccine(s)	206 (51.6)	78 (19.5)	115 (28.8)
Number of doses required to achieve this efficacy	206 (51.6)	106 (26.6)	87 (21.8)
Efficacy of approved vaccine(s) refers to mortality, severe disease, asymptomatic infection, viral transmission, long COVID, or a combination of these outcomes	204 (51.1)	117 (29.3)	78 (19.5)
Efficacy of approved vaccine(s) for specific groups (e.g., over 80 years old, co-morbidities, pregnancy, children)	209 (52.4)	88 (22.1)	102 (25.6)
The potential benefits to patients who have previously been infected with COVID-19	204 (51.1)	83 (20.8)	112 (28.1)
The potential need for future boosters (e.g., annually)	191 (47.9)	67 (16.8)	141 (35.3)
Vaccine eligibility and prioritization			
The order in which groups will be offered an approved vaccine (i.e., the priority list)	201 (50.4)	75 (18.8)	123 (30.8)
The rationale for this priority list	210 (52.6)	111 (27.8)	78 (19.5)
Practicalities of delivering vaccines to patients			
Places where eligible patients will be able to receive an approved vaccine (e.g., GP practice, hospital, mass vaccination center, nursing home, etc.)	207 (51.9)	71 (17.8)	121 (30.3)
The methods to offer an approved vaccine to eligible patients (e.g., patient contacts surgery, surgery contacts patient, etc.)	225 (56.4)	87 (21.8)	87 (21.8)
Vaccine administration			
The ability of patients to choose which approved vaccine they receive (e.g., the one with the highest efficacy)	182 (45.6)	81 (20.3)	136 (34.1)
After-vaccine administration			
The patients should continue to follow public health guidance (e.g., mask-wearing, physical distancing, etc.) after receiving an approved vaccine	201 (50.4)	91 (22.8)	107 (26.8)
Vaccine misinformation			
Instances of vaccine misinformation (including anti-vax conspiracy theories)	175 (43.9)	85 (21.3)	139 (34.8)

Table 3 displays the association between sociodemographic characteristics and vaccine administration in terms of age, specialty, and years of experience. There is a significant relationship between the ability of patients to choose which approved vaccine they receive and each age, specialty, and years of experience of doctors only. About 56.2% is the rate of confidence of doctors aged 35-44 years that the results in the table have shown, whereas only 29.6% of doctors aged ≥45 years were confident with this item with a p-value = 0.010. Primary health care doctors scored a high rate of 49.8%, while secondary health care doctors scored the lowest confidence rate of 39.3% with a p-value <0.001. Doctors with ≤10 years of experience are confident with this item of 47.7%, whereas only 36.8% of doctors with >10 years of experience were confident with this item with p-value = 0.001 (Table 3).

**Table 3:** Association between sociodemographic characteristics and vaccine administration.

The ability of patients to choose which approved vaccine they receive.				
Variables	Confident	Not sure	Not confident	P-value
Age (years)	N = 399 (100%)	N = 399 (100%)	N = 399 (100%)	0.010
25-34	133 (44.5)	70 (23.4)	96 (32.1)	
35-44	41 (56.2)	7 (9.6)	25 (34.2)	
≥45	8 (29.6)	4 (14.8)	15 (55.6)	
Specialty				<0.001
Primary health care	115 (49.8)	62 (26.8)	54 (23.4)	
Secondary health care	48 (39.3)	13 (10.7)	61 (50)	
Others	19 (41.3)	6 (13)	21 (45.7)	
Patient contact				0.685
Everyday	83 (44.9)	41 (22.2)	61 (33)	
Not everyday	99 (46.3)	40 (18.7)	75 (35)	
Years of experience				0.001
≤10 years	154 (47.7)	72 (22.3)	97 (30)	
>10 years	28 (36.8)	9 (11.8)	39 (51.3)	

Table 4 displays the association between sociodemographic characteristics and after-vaccine administration in terms of age, specialty, patient contact, years of experience, and workplace of doctors. There is a significant relationship between this item and all of the categorical variables mentioned above. Starting with the age of doctors, it has been shown that doctors aged 35-44 years are the most confident among all other age groups, with 63% and a p-value <0.001. About 53.7% of primary health care doctors are confident with this item, while only 45.1% of secondary health care doctors are confident with this item, with a p-value = 0.007. About 57.9% is the percentage of doctors who are confident that patients are not necessarily to be contacted every day, with a p-value = 0.001. In terms of doctors' years of experience, results have shown that doctors with ≤10 years of experience are highly confident with 52.6%, whereas only 40.8% of doctors holding >10 years of experience are confident with this item with a p-value = 0.028. South region doctors are highly confident with this item with 62.5%, followed by central region doctors with 57.8% and east region doctors with 54.1% with a p-value <0.001 (Table 4).

**Table 4:** Association between sociodemographic characteristics and after-vaccine administration.

The patients should continue to follow public health guidance (e.g., mask-wearing, physical distancing, etc.) after receiving an approved vaccine				
Variables	Confident	Not sure	Not confident	P-value
Age (years)	N = 399(100%)	N = 399(100%)	N = 399(100%)	<0.001
25-34	149 (49.8)	71 (23.7)	79 (26.4)	
35-44	46 (63)	16 (21.9)	11 (15.1)	
≥45	6 (22.2)	4 (14.8)	17 (63)	
Specialty				0.007
Primary health care	124 (53.7)	49 (21.2)	58 (25.1)	
Secondary health care	55 (45.1)	24 (19.7)	43 (35.2)	
Others	22 (47.8)	18 (39.1)	6 (13)	
Patient contact				0.001
Everyday	77 (41.6)	56 (30.3)	52 (28.1)	
Not everyday	124 (57.9)	35 (16.4)	55 (25.7)	
Years of experience				0.028
≤10 years	170 (52.6)	65 (20.1)	88 (27.2)	
>10 years	31 (40.8)	26 (34.2)	19 (25)	

## Discussion

The results of this study found that the confidence of physicians in the Kingdom of Saudi Arabia in counseling patients about COVID-19 disease and COVID-19 vaccines showed a number of important findings. It was found that most doctors in our study possessed good overall knowledge of the COVID-19 vaccine, which was reflected in the 56.2% rate of confidence of doctors aged 35-44 years. Another crucial finding was that doctors aged 35-44 years are the most confident among all other age groups, with 63% and a p-value <0.001 in administering the vaccine to patients. The distribution, both by gender or age and by professional degree, was well-balanced, and the majority worked in a clinical setting at the time of the survey. This part presents the descriptive and analytical findings of the survey questionnaires. It discusses the result output to ascertain the nature of relationships among the research variables. The latter, discussed in the context of the research questions and objectives of the study, is to determine the reliability and validity of the methodological methods employed in the data collection and processing stages, culminating in the justification of the application of the statistical tools of analysis attested in the following sections on the tabulated finding-results for summarized interpretative discussion. COVID-19 imposed a very hard challenge for the whole world and for doctors and labs in particular to come up with a vaccine that would relief people from the threats they were facing from the pandemic. In this study, 399 Saudi doctors were asked to self-assess their knowledge in counseling about COVID-19 disease and vaccination and to answer a survey of their perspectives in the Kingdom of Saudi Arabia.

In terms of the sociodemographic characteristics of participants, our study showed that the majority of respondents were male doctors, with a percentage of 63.9%, whereas female respondents scored a lower percentage of only 36.1%. About 25-34-year-old respondents were the majority among other age groups, with a percentage of 74.9%. Our study revealed that patient contact was not needed on an everyday basis, with a percentage of 53.6%. This suggests that, even though COVID-19 was a nagging issue, the study scored a majority of male respondent dominance, which can be interpreted in a variety of ways in many supportive studies. Mansour et al. corroborated this finding by pointing out that a global survey found consensus on the amount of care physicians had to provide during the COVID-19 pandemic, particularly when working in unprepared communities or institutions. According to their study, the majority of doctors had a solid general understanding of COVID-19; our study's findings support this. They continued by saying that doctors' main information sources were official government websites. This indicates that doctors have been regularly using trustworthy sources to learn about COVID-19, which is consistent with the high level of knowledge seen [15].

Gender was found to be a predictor of the level of acceptance of the COVID-19 vaccine among physicians and other healthcare workers in Saudi Arabia, according to a different study by Shehata et al [16]. Qattan et al. (2021) found that more males (72.19%) accepted to be vaccinated when available than females, but they did not find any significant difference among different age groups. Two related studies, carried out in Ghana and Egypt, revealed the same results. A noteworthy discovery regarding patient gender was made by Nikic et al. in another study, who reported that the vaccination rate was considerably higher in male patients. Consistent with our findings, a study carried out on a community exhibiting high vaccination compliance rates emphasized that female gender is one of the contributing variables to vaccine non-acceptance against COVID-19. Furthermore, men are more likely to experience more severe COVID-19 infection outcomes and morphologies, according to a meta-analysis by Booth et al., which may help to explain our results. Nonetheless, contrary to what we found, other research has indicated that women are more likely to accept vaccinations [17].

According to Qattan et al. (2021), there has even been evidence of vaccine resistance among healthcare professionals. Despite the absence of significant adverse effects or vaccine-related breakthrough infections, reported symptoms varied across males and females in all age categories. In addition to beliefs, campaign execution, knowledge levels, and attitudes, perceived susceptibility and benefit are significant factors in the intention to vaccinate. These constructions are connected with sociodemographics such as sex, nationality, education, employment in the healthcare industry, and monthly income.

Our study has clearly highlighted very important results in regard to the rate of safety of the vaccine. It was found that the rate of confidence of the vaccine safety is believed to have common side-effects associated with the approved vaccine(s) scored a percentage of 62.7%. Doctors were confident that there is a potential for adverse effects that are not yet seen in vaccine trials with 45.1%. This result stressed the important role of doctors in reassuring people about the importance of the vaccine yet passing this knowledge to patients. This was supported by Alzarea et al., who reported that in randomized control trials, these vaccines reported local (injection site pain, redness, and swelling) and systematic (muscle or joint pain, headache, and fatigue) side effects. The proportion of these side effects varied across the studies, from 50% to 90%. Since existing data on the side effects of COVID-19 vaccines have emerged from health authorities, there is little information on real-world and patient-reported side effects after receiving COVID-19 vaccine doses.

According to our research, 62.7% of respondents felt that the authorized vaccine(s) had common adverse effects. This indicates a high level of confidence in vaccination safety. The findings and conclusions generally corroborate the assertions made by Aldossari [18] that different sectors responded differently to the vaccine's introduction, with some being in favor and others opposed. The majority of participants used medication; however, there were few medical consultations or hospital admissions. Short-term symptoms included weariness, soreness at the vaccination site, fever, chills, and headache, especially during the second haze. Furthermore, vaccination reluctance is a topic of discussion, particularly with adults, as a result of misconceptions about safety and efficacy. To dispel most of these myths, vaccine literacy programs are advised. Despite the Ministry of Health's encouragement of COVID-19 immunizations to preserve community immunity, a substantial percentage of people refused the shots, even after it was made clear how safe, effective, and reliable they were. However, vaccination reluctance has been linked to factors such as age, gender, and nationality; the adequacy of clinical trials; and the identification of adverse effects. On the other hand, vaccination willingness was higher among the elderly, married individuals, foreigners, and those with higher levels of education [18]. 

The mortality, severe illness, asymptomatic infection, viral transmission, prolonged COVID-19 infection, or a combination of these events are considered the efficacy of the approved vaccine(s). Physicians also showed a comparable level of trust in the potential advantages for patients who have already contracted COVID-19, with 51.1% of respondents. These findings are corroborated by Ganesan et al., who reported that the biggest incentive to get vaccinated was the vaccine's safety, with no serious adverse effects, according to a survey on vaccination perception conducted in the United Arab Emirates for the COVID-19 vaccine. Studies are carried out to track the safety of COVID-19 vaccines worldwide because concerns about side effects and vaccine safety influence immunization decisions. According to all of these findings, people's main reason for not getting vaccinated is fear of the negative side effects or events that could occur from the COVID-19 immunization [19].

Further, with regards to the practicalities of delivering vaccines to patients, the methods to offer an approved vaccine to eligible patients (e.g., patient contacts surgery, surgery contacts patient, etc.) were much trusted by doctors with 56.4%. The results showed that the rate of confidence of the ability of patients to choose which approved vaccine they receive (e.g., the one with the highest efficacy) was 45.6% in regard to vaccine administration by doctors. This result is consistent with findings by Lin et al., who stated that it is highly likely that vaccine hesitancy is universal across countries, states, and subgroups (including healthcare providers and parents), so are its determinants-perceived disease or outbreak severity, infection risk, and vaccine safety, effectiveness, and necessity [9]. Influenza vaccination history, trust in the government, and doctor’s recommendations are important facilitators for vaccine confidence and acceptance. These findings are in line with previous research on other vaccines. Nonetheless, increasing daily cases and deaths did not prevent double-digit declines in vaccination intention since its highest point in early April.

Regarding the after-vaccine administration, it was discovered that patients felt confident in the scenario where they should keep up with public health recommendations after obtaining an approved vaccination (50.4%). The majority of people are willing to accept vaccinations because they have faith in government decisions and the healthcare system, according to Mansour et al. This is because they recognize that the risk of COVID-19 is primarily dependent on actions taken by health authorities, medical professionals, and other entities. Healthcare professionals have also been found to be resistant to vaccines. Despite the absence of significant adverse effects or vaccine-related breakthrough infections, reported symptoms varied across males and females in all age categories. In addition to beliefs, campaign execution, knowledge levels, and attitudes, perceived susceptibility and benefit are significant factors in the intention to vaccinate. These are connected with sociodemographics such as sex, nationality, education, employment in the healthcare industry, and monthly income [15].

Our study dealt with a very crucial yet nagging issue, which is the association between sociodemographic characteristics and after vaccine administration in terms of age, specialty, patient contact, years of experience, and the workplace of doctors. The study found that doctors aged 35-44 years are the most confident among all other age groups, with 63% and a p-value <0.001. About 57.9% is the percentage of doctors who are confident that patients are not necessarily to be contacted every day, with a p-value of 0.001. The findings of Nikic et al., who claimed in their publication that their data indicated a strong influence of family variables on patients' decisions to vaccinate, provide strong support for the earlier results. Specifically, compared to patients who lived alone or with multiple household members, those who shared a residence had the highest vaccination rate. Additionally, their results demonstrated that patients were more likely to get vaccinated themselves if they indicated that a family member's influence had a significant role in their decision to finish immunization and if they had family members who had received the COVID-19 vaccine [17]. The authors argue that the high frequency of conspiracy theories regarding COVID-19, which was purposefully introduced into the world to aid in the development of vaccines against it, may be the cause of these disparities in vaccination willingness among various nations. Additionally, the degree of vaccine adoption among individuals, including doctors and other healthcare professionals, is impacted by the dissemination of false information regarding the efficacy and safety of COVID-19 vaccinations, particularly in poor nations [16].

Another important finding the previous study found, which in turn supports our findings of respondents' fear of the after-vaccine administration can be drawn to the most frequent reasons for vaccination unacceptability among the included Egyptian physicians were fear of adverse side effects, as it was reported by more than half of physicians, followed by the short duration of clinical trials performed on the vaccine; then, concerns about the vaccine's safety and efficacy were reported by only one-third of physicians.

Strengths and limitations

The study population is to be considered one of the most important strengths, and 399 participants were included. Doctors from different specializations who came from different parts of Saudi Arabia have participated in this study. Since we were able to include physicians from various professional levels, specializations, and regions of the Kingdom, the results can be considered an accurate representation of medical opinion as of the time the questionnaire was released. This is in line with the good knowledge noted and indicates that physicians have been regularly using trustworthy sources to learn about COVID-19. Additionally, study participants demonstrated significant knowledge in areas related to the causes and therapies of disease. Nevertheless, as with every study, this one had certain shortcomings. One issue we ran into was that the study was only conducted among hospitals in the private sector; the outcomes could have been different if the public hospital had also been involved. Particular difficulties with online surveys include the difficulty of computing response rates, the risk of recollection bias, and the likelihood of non-representative data. In addition, the study was conducted after the COVID-19 vaccine was made legal, which may not have assisted in building the necessary confidence in the vaccine's efficacy. As a result, the results may not accurately reflect the new information that has come to light since the study was conducted. It's also critical to remember that information is constantly changing, so the results shown here reflect the consensus as of the end of 2022. However, it's crucial to keep in mind that some doctors may not have known as much as they should have known at that point about the precise level of confidence due to a lack of understanding.

## Conclusions

The study concluded that most doctors had a good level of confidence and knowledge about the COVID-19 vaccine. They expressed concerns about ensuring accurate information reaches the general population regarding doctors' confidence in the vaccine's safety. The issues regarding doctors' confidence could persist due to the study's focus on private hospital doctors and the potential exacerbation of weaknesses when including doctors from public hospitals in future surveys. To improve the image and confidence among doctors, it is crucial to take positive steps in improving health policies, plans, and programs. Additionally, a comprehensive training plan should be implemented to enhance doctors' knowledge and awareness of vaccine administration. It is important for doctors to have a strong educational background and practical experience in order to administer vaccines with confidence. In addition to their clinical skills, the study recommends that doctors participate more actively in policymaking, especially in times of health emergencies and potential pandemics.
